# Eosinophilic Esophagitis in a 3-Year-Old Girl with Spinal Muscular Atrophy Type 1: The First Reported Case

**DOI:** 10.3390/pediatric17040080

**Published:** 2025-07-28

**Authors:** Aleksandra Marzec, Elżbieta Jarocka-Cyrta, Marta Ruskań-Bakun

**Affiliations:** 1 Department of Clinical Pediatrics, Faculty of Medicine, University of Warmia and Mazury in Olsztyn, 10-561 Olsztyn, Poland; elzbieta.jarocka@uwm.edu.pl; 2 Department of Pediatrics, Gastroenterology and Nutrition, Provincial Specialist Children’s Hospital in Olsztyn, 10-561 Olsztyn, Poland; 3 Nicolaus Copernicus Superior School, Doctoral School, 00-695 Warsaw, Poland; 4 Department of Pathomorphology and Forensic Medicine, Faculty of Medicine, University of Warmia and Mazury in Olsztyn, 10-561 Olsztyn, Poland; ruskan@wp.pl

**Keywords:** spinal muscle atrophy type 1, eosinophilic esophagitis, pediatrics

## Abstract

**Background:** Spinal muscular atrophy type 1 (SMA1) is a severe neuromuscular disorder characterized by progressive muscle weakness and atrophy, including the muscles of the oral cavity and esophagus. Eosinophilic esophagitis (EoE), a chronic, allergic disease, presents with eosinophilic infiltration of the esophagus, leading to esophageal dysmotility. Feeding difficulties may occur in both conditions. So far, the coexistence of EoE and SMA1 has not been described; we present the first such case. **Case presentation:** The patient was a girl with SMA1 diagnosed shortly after birth, treated with nusinersen and onasemnogene abeparvovec, and fed a standard industrial diet through a gastrostomy. In her second year of life, she developed increasing symptoms: distress during feeding, regurgitation, vomiting, and weight loss. She was treated with proton pump inhibitors without clinical improvement. Gastroscopy was performed, revealing superficial epithelial damage with bleeding in the proximal esophagus. Histopathology showed chronic inflammation with up to 150 eosinophils per high-power field, microabscesses, spongiosis, and basal layer hypertrophy. The girl was diagnosed with EoE. Her diet was switched from a standard industrial formula to an amino acid-based formula, which led to marked clinical improvement, the resolution of symptoms, and appropriate weight gain. **Conclusions:** This case report highlights the challenges of diagnosing EoE in SMA1 patients and emphasizes the need for multidisciplinary approaches and further investigation of allergic manifestations in SMA1 patients.

## 1. Introduction

Spinal muscular atrophy type 1 (SMA1) is an autosomal recessive neuromuscular disease caused by a homozygous mutation or deletion in the survival motor neuron 1 gene (SMN1). It has an estimated incidence of 6 to 10 per 100,000 live births, and the carrier frequency in the Caucasian population is at 1 in 45 individuals [1,2]. SMA1 is characterized by progressive muscle atrophy from the first days of life. Respiratory muscle weakness, cough impairment, and recurrent pulmonary infections result in respiratory failure. The neuromuscular dysfunction also affects the muscles of the mouth and esophagus, resulting in feeding difficulties, dysphagia, vomiting, aspiration, and malnutrition. In most cases, enteral feeding through a gastrostomy is necessary [3]. Motor function in infants with SMA type 1 is assessed using the CHOP INTEND (Children’s Hospital of Philadelphia Infant Test of Neuromuscular Disorders), a validated clinical scale specifically designed for evaluating motor skills in children with severe neuromuscular disorders. The total score ranges from 0 to 64 points, with lower scores indicating more severe motor impairment. A score below 20 points is typically associated with minimal spontaneous movement and a poor prognosis, while scores above 40 suggest improved motor function and are often observed following early therapeutic intervention [4]. The introduction of newborn screening programs has significantly improved early detection, enabling timely therapeutic interventions that have translated into markedly enhanced survival rates and quality of life for affected children [5].

Eosinophilic esophagitis (EoE) is a chronic condition in which eosinophils infiltrate the esophageal wall, causing inflammation, tissue remodeling, and dysmotility. In infants, clinical symptoms are nonspecific and include reluctance to eat, regurgitation, and vomiting, which can lead to failure to thrive [6]. The population-based prevalence of EoE in children is approximately 19.1 cases per 100,000 children per year, with significant geographic variation—ranging from 2.3 per 100,000 in Denmark to 50.5 per 100,000 in the United States [7,8,9]. The incidence and recognition of EoE have steadily increased over the past decades, particularly in the pediatric population. This trend is attributed to not only a genuine rise in disease occurrence but also greater clinical awareness and wider use of endoscopic and histopathological diagnostics. EoE is more frequently diagnosed in boys, with a male–female ratio of approximately 3:1, and shows a strong association with atopic conditions such as asthma, allergic rhinitis, atopic dermatitis, and food allergies [10,11]. The higher rate of EoE was described in patients with connective tissue disorders and esophageal atresia [12,13]. The association of EoE and SMA1 represents an emerging clinical challenge, as similar symptoms may hinder timely diagnosis and appropriate management, highlighting the need for increased clinical awareness and further research to elucidate the potential association between these conditions. The coexistence of EoE and SMA1 has not yet been described; we present the first such case.

## 2. Case Presentation

The patient is a girl from a first pregnancy, born at term, via spontaneous delivery, with an APGAR score of 9/10. She was diagnosed with SMA1 in the first month of life, treated with nusinersen from the third month of life, and received onasemnogene abeparvovec in the seventh month of life. At birth, she was assessed at 13/56 points on the CHOP INTEND scale. Following pharmacological treatment, her score improved to 54/56. She required nocturnal respiratory support via BiPAP. Due to severe dysphagia, a gastrostomy was performed at age 7 months. The patient was fed a standard, commercial, age- appropriate 1 kcal/mL formula with good tolerance. In the second year of life, the parents observed increasing symptoms—distress during feeding; regurgitation; vomiting; periodically with blood strips; and weight loss. At the time of admission, her general condition was stable. The patient was conscious, in a supine position, and made eye contact. Physical examination revealed poorly developed subcutaneous tissue (body weight < third percentile), a bell-shaped chest, and a bitemporally flattened skull. Neurological examination showed reduced spontaneous limb movements (she could lift both upper and lower extremities), generalized hypotonia, decreased muscle strength, and absent tendon and periosteal reflexes in upper and lower limbs. She could rotate independently along her longitudinal axis, maintained a sitting position when placed, and attempted to assume a quadruped (all-fours) position.

**Figure 1:**
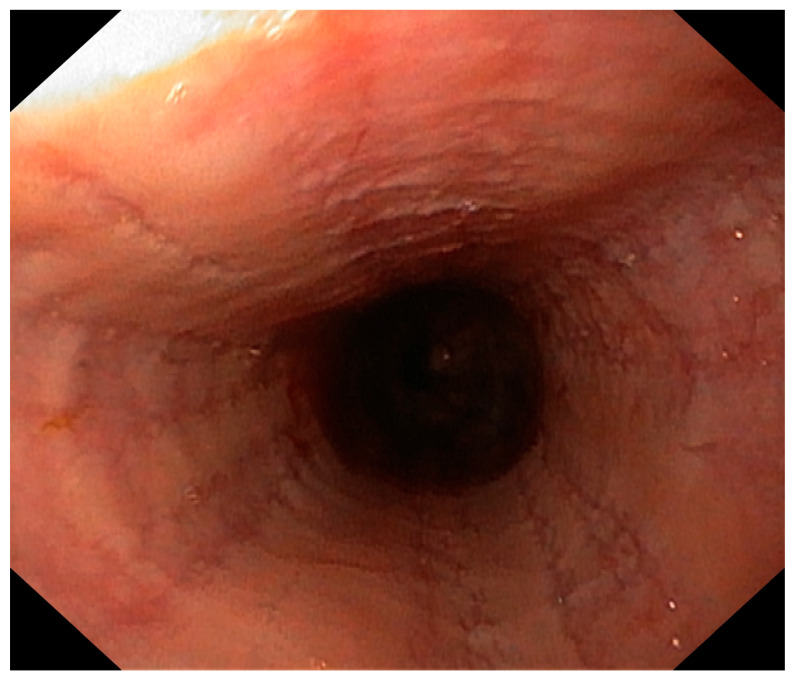
Endoscopic appearance of the esophagus.

**Figure 2:**
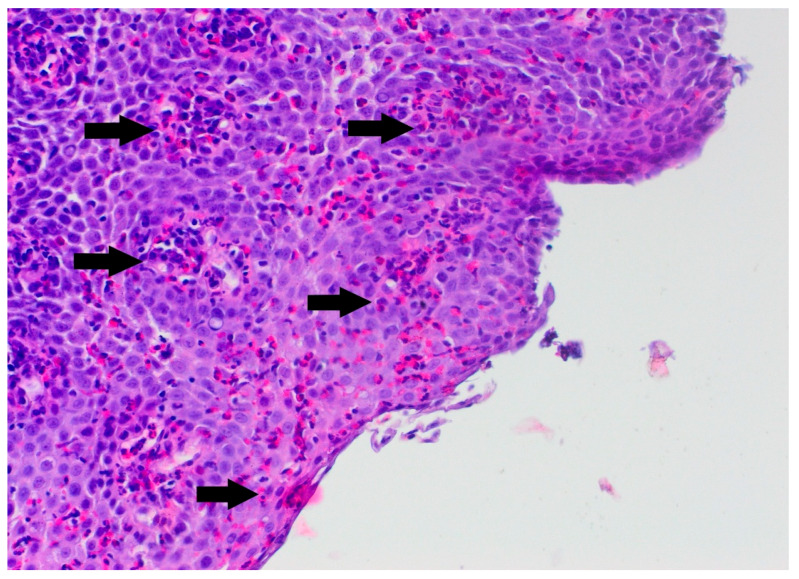
The biopsy specimen shows numerous eosinophils in the stratified epithelium of the esophagus. Eosinophils in eosinophilic esophagitis are typically found in greater numbers closer to the epithelial surface rather than near the base. Arrows indicate eosinophils (hematoxylin and eosin stain, ×200).

**Figure 3:**
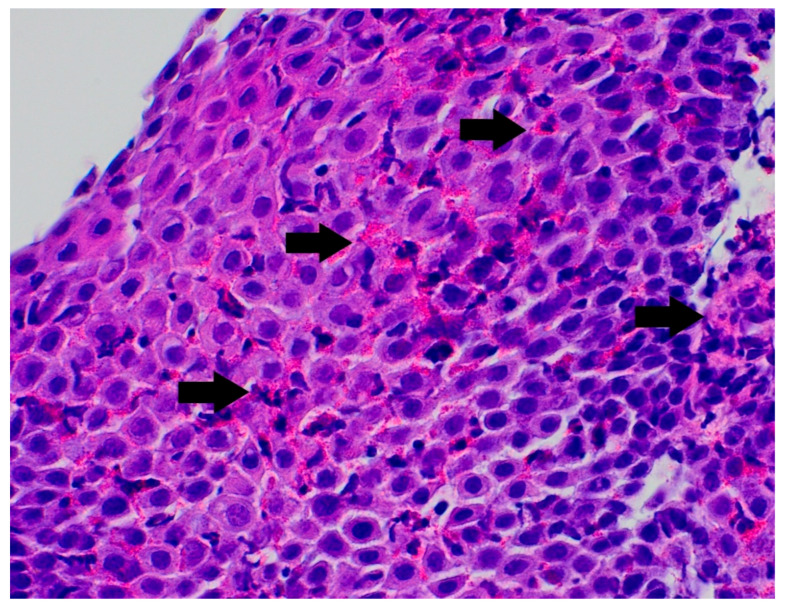
The distal esophageal biopsy specimen shows several degranulated eosinophils. This feature helps confirm the diagnosis of eosinophilic esophagitis. Arrows indicate eosinophils (hematoxylin and eosin stain, ×400).

**Figure 4:**
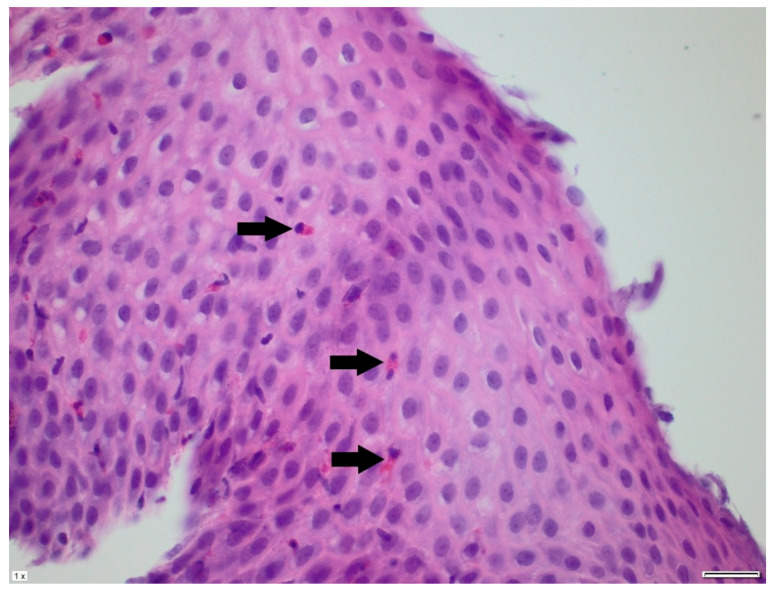
A sample taken from the patient after treatment. Single, dispersed eosinophils are observed in the esophageal epithelium, with fewer than 15 present in a high-power field. Arrows indicate eosinophils.

In the differential diagnosis, esophageal motility disorders related to SMA1, food allergy, and side effects of the medications used in SMA1 treatment were considered. She was treated with proton pump inhibitors and prokinetic agents without clinical improvement. Her diet was subsequently changed from a standard formula to a protein hydrolysate formula, also without effect. Due to the persistence of symptoms, an upper gastrointestinal endoscopy was performed. It revealed superficial epithelial damage of the esophagus with bleeding in the proximal part (Figure 1). Histopathological examination showed chronic inflammatory infiltration of the esophagus, with up to 150 eosinophils per high-power field, scattered and forming microabscesses, spongiosis, and basal layer hypertrophy (Figure 2 and Figure 3). She was diagnosed with EoE. In laboratory tests, specific IgE antibodies against bovine serum albumin (22.75 kU/L), casein (2.72 kU/L), beta-lactoglobulin (9.45 kU/L), and alpha-lactalbumin (6.45 kU/L) from cow’s milk were detected, but no clinical IgE-dependent reactions were presented. Otherwise, laboratory tests revealed no significant abnormalities (results are presented in Table 1). An amino acid-based formula was applied in feeding, resulting in clinical enhancement and improved weight gain. Follow-up gastroscopy revealed resolved esophageal damage with a decreased eosinophil count per high-power field in histopathology (Figure 4).

## 3. Discussion

EoE is a chronic inflammatory antigen-mediated disease, with a varying clinical presentation and increasing recognition. It can occur at any age, including early childhood. The diagnosis is based on clinical symptoms of esophageal dysfunction and an increased number of eosinophiles in the esophageal wall (≥15 per high-power microscopy). During the diagnostic process, it is crucial to exclude other diseases causing eosinophilia, including drug-induced eosinophilia [6,14]. The overall clinical picture depends on the age of the presentation. Infants and toddlers present with nonspecific symptoms, such as food refusal, vomiting, and regurgitation, which may lead to failure to thrive [15]. The clinical manifestations of EoE can mimic those of GERD, which may delay the appropriate diagnosis and treatment. The management strategies of EoE are based on pharmacological therapies, including proton pump inhibitors, topical corticosteroids, and dietary approaches with food elimination or elemental diets [14].

Spinal muscular atrophy is a severe autosomal recessive neuromuscular disease. It is caused by a homozygous deletion in the SMN1 gene responsible for producing the SMN protein, leading to the degeneration of the neurons of the anterior spinal cord nuclei and progressive impairment of skeletal muscles. This condition has four distinct types, categorized based on the severity of the symptoms and the age at which they manifest. The most severe variant is SMA1, characterized by its early onset—before age 6 months. Symptoms observed in SMA1 primarily stem from a progressive decline in muscle strength [3]. However, this condition is also recognized as a multisystemic disease with an overall dysfunction in peripheral tissues and organs, which in combination leads to significant disturbances in the patient’s development and functioning [16]. Weakness and wasting of the limbs result in failure to achieve milestones—head control, sitting, standing, walking, hypotonia, and areflexia. Diminished strength of the intercostal muscles may lead to respiratory distress and pulmonary complications. The gastrointestinal symptoms of SMA1 are associated with bulbar muscle dysfunction and esophageal wall impairment, resulting in intolerance to bolus feeding, poor motility, and difficulties with swallowing; these may overlap with EoE symptoms. Patients with SMA1 often require gastrostomy and Nissen fundoplication, as this can lead to enhanced nutritional well-being and a reduction in aspiration [17].

SMA1 treatment is based on a multidisciplinary approach, including nutritional, gastrointestinal, and orthopedic management, and treatment influencing the genes involved in SMA; gene replacement therapy—onasemnogene abeparvovec; and substances that directly increase gene expression—nusinersen and risdiplam [18].

Before the introduction of specific gene therapies, SMA1 was among the leading genetic causes of infant mortality. The median survival was 7.4 months, with a range of 3–56 months. The best treatment outcomes may be obtained when intervention is initiated during the disease’s early stages. Administering a drug at the presymptomatic stage can prevent the full development of symptoms [19]. Historically, most children diagnosed with SMA1 did not survive long enough to experience typical allergic symptoms, and the presence of underlying disease complications could mask the clinical presentation of allergies. Currently, no data exist on the prevalence and characteristics of other allergic diseases in children with SMA. Moreover, no reports exist on whether the type of treatment influences the occurrence of EoE in these patients, although this issue warrants particular attention.

The available literature describes only two cases of EoE in patients with SMA type 2. SMA type 2 develops between ages 6 and 18 months, and is characterized by a milder clinical presentation than SMA type 1 [2]. A study conducted by Fuller presents two cases of comorbidity involving SMA type 2 and EoE [20]. Both patients had symptoms similar to those of our patient, including weight loss. In both cases, pharmacological treatment was used with only moderate improvement. In one patient, dietary management with a protein hydrolysate formula was introduced, leading to significant clinical recovery.

Our patient exhibited symptoms of EoE typical for her pediatric age group, prominently featuring vomiting. GERD was excluded due to the lack of response to proton pump inhibitor therapy. Notably, these manifestations were observed several months post-initiation of nusinersen treatment. Most adverse effects associated with nusinersen can be attributed to the lumbar puncture procedure, the primary method of administering the drug. Vomiting was identified as the predominant adverse event observed within a 72 h post-lumbar puncture time frame [21]. Moreover, in this case, no temporal association between the medication intake and symptom onset was discerned by the parents, which also suggests that vomiting was not a medication side effect.

EoE is an IgE-independent allergy with cow’s milk proteins being the most common allergen. Some patients have positive specific IgE to food allergens; however, this does not affect the diagnosis or treatment of EoE. In the described case, food-specific IgE testing revealed positive results for cow’s milk proteins, confirming sensitization. While such testing can support allergy evaluation in patients with EoE, its low predictive value for identifying causative foods limits its clinical utility [22]. In the first months of life, the child was fed cow’s milk formula and was exposed to cow’s milk allergens. The commercial formula introduced for enteral feeding also contained cow’s milk proteins. During this period, sensitization may have occurred due to esophageal contact with cow’s milk allergens during episodes of gastroesophageal reflux. Gastroesophageal reflux is very common in children with SMA.

Dietary therapy is a cornerstone of EoE management in children and has proven efficacy in achieving histological remission. Current guidelines recommend an individualized approach: starting with a one-food elimination diet, typically removing cow’s milk, and escalating to a four-food elimination diet (eliminating cow’s milk, wheat, egg, and soy/legumes) or a six-food elimination diet (eliminating cow’s milk, wheat, egg, soy/legumes, nuts, and seafood) based on clinical and histologic responses. An elemental diet based on amino acids and completely free of intact proteins should be used only as rescue therapy [14]. Since the patient was already receiving an industrial formula via gastrostomy, an elemental diet was introduced as a first choice rather than a stepwise elimination diet.

## 4. Conclusions

This report presents the first documented case of EoE in a patient with SMA1, highlighting a previously unrecognized clinical association that may pose diagnostic and therapeutic challenges. The overlap in gastrointestinal manifestations, including vomiting, regurgitation, and feeding difficulties, underscores the need for heightened clinical vigilance in differentiating EoE from complications inherent to SMA1. The identification and successful treatment of eosinophilic EoE led to the resolution of symptoms. This observation raises the question of whether the coexistence of EoE and SMA 1 is incidental, or if there may be an underlying predisposition in this patient population. Further studies are needed to explore this potential association.

## Figures and Tables

**Table 1:** Laboratory test results, WBC—white blood cell count, NEU #—neutrophil count, LYM #—lymphocyte count, EOS #—eosinophil count, CRP—C-reactive protein, AST—aspartate aminotransferase, ALT—alanine aminotransferase, sIgE—specific immunoglobulin E.

Parameter	Result	Reference Range	Unit
Red blood cells	3.8	3.6–5.1	×10^6^/mm^3^
Hemoglobin	11.3	10.2–14.2	g/dL
Hematocrit	34.8	31.0–40.5	%
Platelets	211	140–440	×10^3^/mm^3^
WBC	15.3	5.5–15.5	×10^3^/mm^3^
NEU #	4.65	1.5–8.5	×10^3^/mm^3^
LYM #	10.02	2.0–8.0	×10^3^/mm^3^
EOS #	0.28	0.02–0.65	×10^3^/mm^3^
CRP (C-reactive protein)	0.24	<0.50	mg/dL
Albumin	4.0	3.8–5.4	g/dL
AST	42	<56	U/L
ALT	17	<39	U/L
sIgE Bovine serum albumin	22.75	<0.35	kU/L
sIgE Casein	2.72	<0.35	kU/L
sIgE Beta-lactoglobulin	9.45	<0.35	kU/L
sIgE Alpha-lactalbumin	6.45	<0.35	kU/L

## Data Availability

No new data were created or analyzed in this study. Data sharing is not applicable to this article.

## Abbreviations

The following abbreviations are used in this manuscript:

SMA 1	spinal muscular atrophy type 1
EoE	eosinophilic esophagitis
GI	gastrointestinal
GERD	gastroesophageal reflux disease
SMN 1	survival motor neuron 1 gene
CHOP INTEND	Children’s Hospital of Philadelphia Infant Test of Neuromuscular Disorders
SMA 2	spinal muscular atrophy type type 2
EHF	extensively hydrolyzed protein formula
AAF	amino acid-based formulas
